# Effects on Coronary Heart Disease of Increasing Polyunsaturated Fat in Place of Saturated Fat: A Systematic Review and Meta-Analysis of Randomized Controlled Trials

**DOI:** 10.1371/journal.pmed.1000252

**Published:** 2010-03-23

**Authors:** Dariush Mozaffarian, Renata Micha, Sarah Wallace

**Affiliations:** 1 Division of Cardiovascular Medicine and Channing Laboratory, Department of Medicine, Brigham and Women's Hospital and Harvard Medical School, Boston, Massachusetts, United States of America; 2 Department of Epidemiology, Harvard School of Public Health, Boston, Massachusetts, United States of America; 3 Department of Nutrition, Harvard School of Public Health, Boston, Massachusetts, United States of America; Vrije Universiteit Amsterdam, Netherlands

## Abstract

**Background:**

Reduced saturated fat (SFA) consumption is recommended to reduce coronary heart disease (CHD), but there is an absence of strong supporting evidence from randomized controlled trials (RCTs) of clinical CHD events and few guidelines focus on any specific replacement nutrient. Additionally, some public health groups recommend lowering or limiting polyunsaturated fat (PUFA) consumption, a major potential replacement for SFA.

**Methods and Findings:**

We systematically investigated and quantified the effects of increased PUFA consumption, as a replacement for SFA, on CHD endpoints in RCTs. RCTs were identified by systematic searches of multiple online databases through June 2009, grey literature sources, hand-searching related articles and citations, and direct contacts with experts to identify potentially unpublished trials. Studies were included if they randomized participants to increased PUFA for at least 1 year without major concomitant interventions, had an appropriate control group, and reported incidence of CHD (myocardial infarction and/or cardiac death). Inclusions/exclusions were adjudicated and data were extracted independently and in duplicate by two investigators and included population characteristics, control and intervention diets, follow-up duration, types of events, risk ratios, and SEs. Pooled effects were calculated using inverse-variance-weighted random effects meta-analysis. From 346 identified abstracts, eight trials met inclusion criteria, totaling 13,614 participants with 1,042 CHD events. Average weighted PUFA consumption was 14.9% energy (range 8.0%–20.7%) in intervention groups versus 5.0% energy (range 4.0%–6.4%) in controls. The overall pooled risk reduction was 19% (RR = 0.81, 95% confidence interval [CI] 0.70–0.95, *p* = 0.008), corresponding to 10% reduced CHD risk (RR = 0.90, 95% CI = 0.83–0.97) for each 5% energy of increased PUFA, without evidence for statistical heterogeneity (Q-statistic *p* = 0.13; I^2^ = 37%). Meta-regression identified study duration as an independent determinant of risk reduction (*p* = 0.017), with studies of longer duration showing greater benefits.

**Conclusions:**

These findings provide evidence that consuming PUFA in place of SFA reduces CHD events in RCTs. This suggests that rather than trying to lower PUFA consumption, a shift toward greater population PUFA consumption in place of SFA would significantly reduce rates of CHD.

*Please see later in the article for the Editors' Summary*

## Abstract 2

Dariush Mozaffarian and colleagues conduct a systematic review and meta-analysis to investigate the effect of consuming polyunsaturated fats in place of saturated fats for lowering the risk of coronary heart disease.

## Editors' Summary

**Background:**

Coronary heart disease (CHD) is the leading cause of death among adults in developed countries. It is caused by disease of the coronary arteries, the blood vessels that supply the heart with oxygen and nutrients. With age, inflammatory deposits (atherosclerotic plaques) coat the walls of these arteries and restrict the heart's blood supply, causing angina (chest pains that are usually relieved by rest), shortness of breath, and, if these plaques rupture or break, heart attacks (myocardial infarctions), which can reduce the heart's function or even be fatal. The key risk factors for CHD are smoking, physical inactivity, and poor diet. Blood cholesterol levels are altered by consuming dietary fats. There are three main types of dietary fats—“saturated” fatty acids (SFA) and unsaturated fatty acids; the latter can be “mono” unsaturated (MUFA) or “poly” unsaturated (PUFA). Eating SFA-rich foods (for example, meat, butter, and cheese) increases the amount of LDL-C in the blood but also increases HDL-C (the “good” cholesterol) and decreases triglycerides. Eating foods that are rich in unsaturated fatty acids (for example, vegetable oils and fatty fish) decreases the amount of LDL-C and triglycerides in the blood and also raises HDL-C.

**Why Was This Study Done?:**

Because of the connection between eating SFA and high blood LDL-C levels, reduced SFA consumption is recommended as a way to avoid CHD. However, the evidence from individual randomized controlled trials that have studied CHD events (such as heart attacks and CHD-related deaths) have been mixed and could not support this recommendation. Furthermore, dietary recommendations to reduce SFA have generally not specified any replacement, i.e., whether SFA should be replaced with carbohydrate, protein, or unsaturated fats. Because of their beneficial effects on blood LDL-C and HDL-C levels, PUFA could be one important replacement for SFA, but, surprisingly, some experts argue that eating PUFA could actually increase CHD risk. Consequently, some guidelines recommend that PUFA consumption should be limited or even reduced. In this systematic review (a study that uses predefined criteria to identify all the research on a specific topic) and meta-analysis (a statistical method for combining the results of several studies) of randomized controlled trials, the researchers assess the impact of increased PUFA consumption as replacement for SFA on CHD events.

**What Did the Researchers Do and Find?:**

The researchers' search of the published literature, “grey” literature (doctoral dissertations, technical reports, and other documents not printed in books and journals), and contacts with relevant experts identified eight trials in which participants were randomized to increase their PUFA intake for at least a year and in which CHD events were reported. 1,042 CHD events were recorded among the 13,614 participants enrolled in these trials. In their meta-analysis, the researchers found that on average the consumption of PUFA accounted for 14.9% of total energy intake in the intervention groups compared with only 5% of total energy intake in the control groups. Participants in the intervention groups had a 19% reduced risk of CHD events compared to participants in the control groups. Put another way, each 5% increase in the proportion of energy obtained from PUFA reduced the risk of CHD events by 10%. Finally, the researchers found that the benefits associated with PUFA consumption increased with longer duration of the trials.

**What Do These Findings Mean?:**

These findings suggest that the replacement of some dietary SFA with PUFA reduces CHD events. Because the trials included in this study looked only at replacing SFA with PUFA, it is not possible from this evidence alone to distinguish between the benefits of reducing SFA and the benefits of increasing PUFA. Furthermore, the small number of trials identified in this study all had design faults, so the risk reductions reported here may be inaccurate. However, other lines of evidence (for example, observational studies that have examined associations between the fat intake of populations and their risk of CHD) also suggest that consumption of PUFA in place of SFA reduces CHD risk. Thus, in the light of these findings, future recommendations to reduce SFA in the diet should stress the importance of replacing SFA with PUFA rather than with other forms of energy, and the current advice to limit PUFA intake should be revised.

**Additional Information:**

Please access these Web sites via the online version of this summary at http://dx.doi.org/10.1371/journal.pmed.1000252.

- The American Heart Association provides information about all aspects of coronary heart disease for patients, caregivers, and professionals, including advice on dietary fats (in several languages)
- The UK National Health Service Choices Web site provides information about coronary heart disease
- Eatwell, a resource provided by the UK Food Standards Agency, gives advice on all aspects of healthy eating, including fat consumption
- MedlinePlus provides links to further resources on coronary heart disease and on cholesterol (in English and Spanish)

## Introduction

Reduction in saturated fatty acid (SFA) consumption is traditionally a major focus of dietary recommendations to reduce coronary heart disease (CHD) risk. However, effects of such a strategy on clinical CHD events are surprisingly poorly established in both randomized controlled trials (RCTs) [1]–[8] and prospective cohort studies [9]. Prior meta-analyses of RCTs have either studied the effects of very heterogeneous dietary fat interventions on very heterogeneous combinations of cardiovascular outcomes [10], or studied effects of dietary fat interventions on intermediate risk markers, such as blood lipids [11]. Furthermore, although dietary guidelines often recommend reduction in SFA consumption, such guidelines often do not highlight any specific nutrient as preferable for replacing SFA in the diet [12]–[14], implying that any macronutrient replacement (unsaturated fats, carbohydrate, or protein) will produce similar effects.

Consumption of polyunsaturated fatty acids (PUFA) lowers the total∶high-density lipoprotein cholesterol (TC∶HDL-C) ratio, perhaps the best single lipid predictor of CHD risk [15], to a greater extent than carbohydrate or any other major class of fatty acids [11]. PUFA consumption may also improve insulin resistance [16],[17] and reduce systemic inflammation [18]–[20]. These effects on risk factors suggest that PUFA may be an ideal replacement for SFA in the population. However, surprisingly, some scientists and organizations argue that consumption of n-6 PUFA, by far the predominant dietary PUFA, will actually increase CHD risk and have recommended reduced consumption [21]–[23], and the Institute of Medicine recommends only a relatively modest range of 5%–10% energy (%E) consumption from PUFA [24], limiting its plausibility as a meaningful replacement for SFA. Several controlled intervention trials have evaluated whether increasing PUFA consumption, as replacement for SFA, impacts risk of CHD events but results of these trials have been inconsistent, with the majority of studies demonstrating no significant benefits [1]–[8]. Thus, the demonstration of whether replacing SFA with PUFA affects CHD outcomes and, if so, the direction and magnitude of this effect are surprisingly understudied matters of scientific and public health importance. To investigate and quantify this effect, we performed a systematic review and meta-analysis of randomized controlled clinical trials that assessed the impact of increased PUFA consumption, as replacement for SFA, on CHD endpoints.

## Methods

We followed the Quality of Reporting of Meta-analyses (QUOROM – now PRISMA (http://www.prisma-statement.org/)) [25] guidelines throughout the design, implementation, analysis, and reporting of this meta-analysis (see Text S1 for PRISMA Statement).

### Search Strategy

We searched for all RCTs that randomized adults to increased total or n-6 PUFA consumption for at least 1 year without other major concomitant interventions (e.g., blood pressure or smoking control, other multiple dietary interventions, etc.), had an appropriate control group without this dietary intervention, and reported (or had obtainable from the authors) sufficient data to calculate risk estimates with standard errors for effects on occurrence of “hard” CHD events (myocardial infarction, CHD death, and/or sudden death). Studies were excluded if they were observational or otherwise nonrandomized; tested mainly n-3 (rather than total or n-6) PUFA interventions or evaluated only intermediate (e.g., lipid levels) or “soft” (e.g., angina) CHD endpoints; or were commentaries, reviews, or duplicate publications from the same study. We did not restrict to primary or secondary prevention trials, but included this as a prespecified factor for assessment of heterogeneity. We included both feeding trials and trials that utilized dietary advice; for both designs, the average change in PUFA consumption was assessed. Searches were performed of literature published through June 2009 using MEDLINE, Embase, AGRIS, AMED, HMIC, PsycINFO, Cochrane library, Web of Knowledge, CABI, CINAHL, conference abstracts (Zetoc), Faculty of 1,000, grey literature sources (SIGLE), related articles, and hand-searching of reference lists. Authors and experts were also directly contacted to identify potentially unpublished trials and, when necessary, request missing data or clarify methods or results.

A full list of search terms for all databases is available (see Text S2 for Protocol). For example, for MEDLINE, search terms were (“Fatty Acids, Omega-6”[Mesh] OR “unsaturated fatty acid”[tiab] OR “unsaturated fatty acids”[tiab] OR “unsaturated fat”[tiab] OR “unsaturated fats”[tiab] OR “polyunsaturated fatty acid”[tiab] OR “polyunsaturated fatty acids”[tiab] OR “polyunsaturated fat”[tiab] OR “polyunsaturated fats”[tiab] OR “omega-6”[tiab] OR “linoleic”[tiab] OR “octadecadienoic acid”[tiab] OR “safflower oil”[tiab] OR “sesame oil”[tiab] OR “soybean oil”[tiab] OR “soyabean oil”[tiab] OR “corn oil”[tiab]) AND (“cardiovascular diseases”[Mesh] OR “cardiovascular disease”[tiab] OR “cardiovascular diseases”[tiab] OR “heart disease”[tiab] OR “heart diseases”[tiab] OR “myocardial infarction”[tiab] OR “myocardial infarctions”[tiab] OR “heart attack”[tiab] OR “heart attacks”[tiab] OR “sudden death”[tiab] OR “sudden deaths”[tiab] OR “coronary syndrome”[tiab]) and NOT (“Fatty Acids, Omega-3”[Mesh] OR “omega-3”[tw] OR “n-3”[tw] OR “stroke”[tiab] OR “strokes”[tiab] OR “cerebrovascular accident”[tiab] OR “cerebrovascular accidents”[tiab] OR “Case Reports”[Publication Type]); limited to humans, adults, and clinical trials or RCTs; through June 2009 without other date or language limitations. For other databases, search terms followed similar concepts with variations based on the database structure.

### Selection of Articles

**Figure 1:**
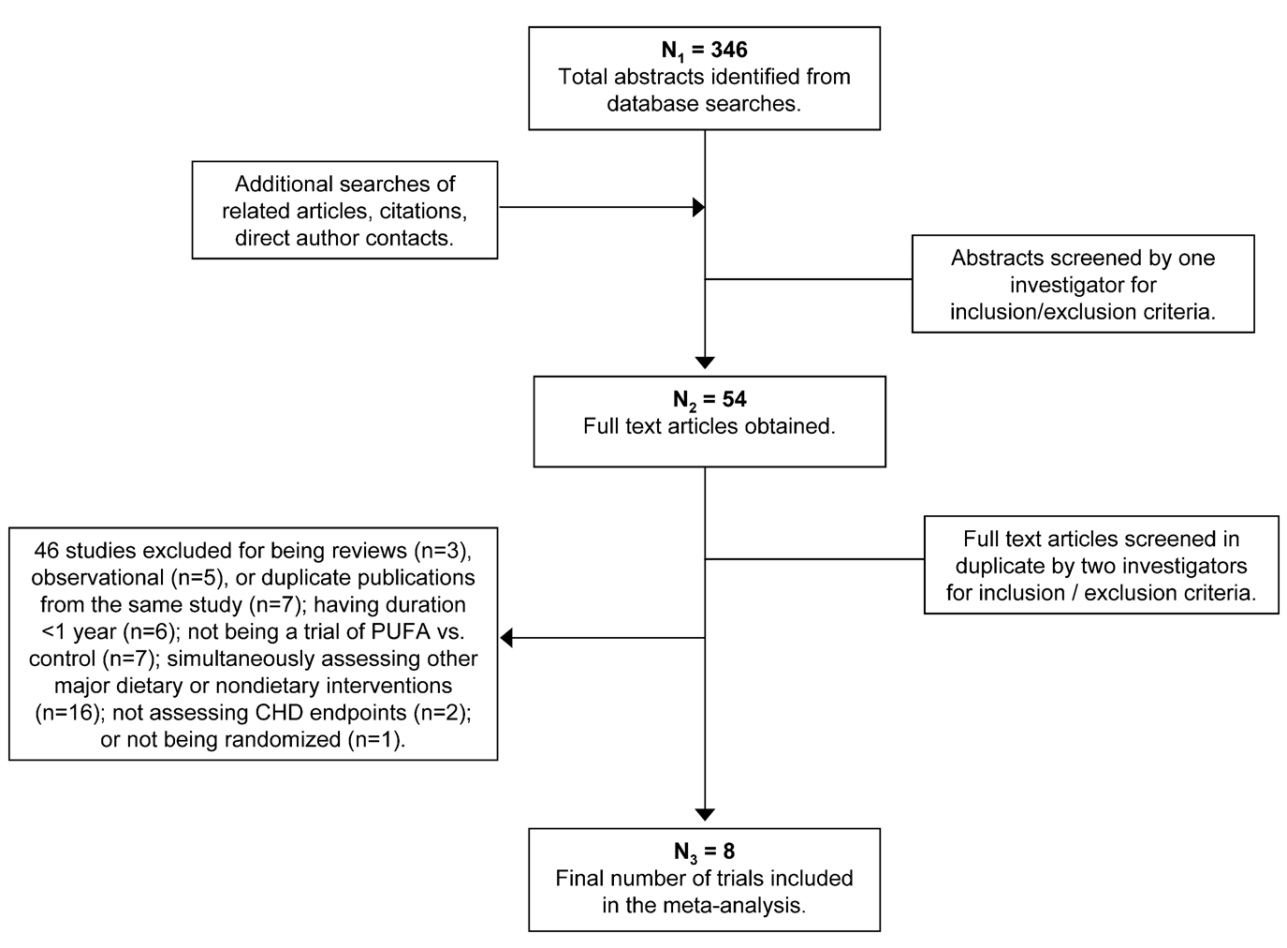
Results of the systematic search strategy and study selection process.

Of 346 identified articles, 290 were excluded based upon review of the title and abstract (Figure 1). Full texts of the remaining 54 manuscripts were independently assessed in duplicate by two investigators to determine inclusion/exclusion. Forty-six studies were excluded because they did not meet inclusion and exclusion criteria (Table S1). The independent duplicate inclusion/exclusion adjudications were 96% concordant on initial comparison. The rare differences were resolved by group consultation among all investigators, with unanimous consensus.

### Data Extraction

For each of the final identified trials, data were extracted independently and in duplicate by two investigators, including years the study was performed and reported, population characteristics, control and intervention diets, duration of follow-up, numbers and types of first CHD events during follow-up, risk ratios (RRs), and standard errors (SEs) of these estimates. When the latter were not available, they were directly calculated using binomial tests of proportions, given that most studies reported RRs rather than incidence rates; stronger findings were seen if SEs were directly calculated using person-time at risk for two reports using incidence rates (unpublished data). Differences in data extracted or quality assessment scores between investigators were very unusual and were resolved by consensus. Several different criteria have been proposed for judging quality of randomized trials in meta-analyses, although the validity and utility of different quality scores has been debated [26]. We assessed study quality using the validated Jadad scale [27], which includes criteria relating to randomization, blinding, and withdrawals and dropouts that are together summed to generate an overall quality score between 0 and 5. Following prior precedent [27], quality scores of 0–2 indicated lower-quality trials, and quality scores of 3–5 indicated higher-quality trials.

### Statistical Analysis

The overall pooled effect was calculated using random effects meta-analysis, which accounts for heterogeneity in treatment effects among studies, using the methods of Dersimonian and Laird [28] with inverse-variance (SE) weighting. Heterogeneity between studies was evaluated using the Dersimonian and Laird Q-statistic, the I^2^ statistic, and meta-regression [28],[29]. Potential for publication bias was assessed by visually inspecting a funnel plot of the treatment effect versus SE [30] and statistically using the Begg adjusted-rank correlation test [31]. Prespecified potential sources of heterogeneity were explored using stratified inverse-variance weighted random effects meta-analysis and inverse-variance weighted metaregression, including trial duration (< or ≥ median for all trials), study population (primary versus secondary prevention), and overall quality score (0–2 versus 3–5). We also performed post-hoc secondary analyses for CHD mortality alone and total mortality, as well as based on selected study characteristics, such as enrollment design (excluding trials with open enrollment), extent of blinding, and type of dietary intervention (provision of meals versus dietary advice). Analyses were performed using STATA 10.1 (College Station, TX), with two-tailed alpha <0.05.

## Results

**Figure 2:**
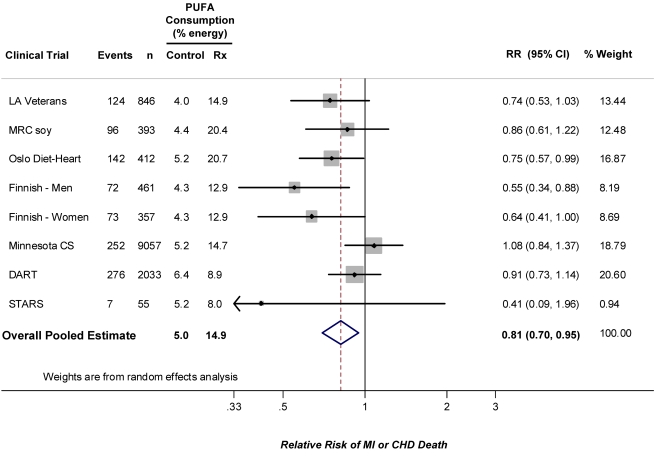
Meta-analysis of RCTs evaluating effects of increasing PUFA consumption in place of SFA and occurrence of CHD events.

The identified RCTs included a total of 1,042 CHD events among 13,614 participants (Table 1) [1]–[8],[32]–[34]. Average PUFA consumption ranged from 4.0%E to 6.4%E (weighted mean 5.0%E) in the control groups and from 8.0%E to 20.7%E (weighted mean 14.9%E) in the intervention groups. Diet was assessed in the majority of trials by either direct analysis of provided foods or by multiple-day weighed diet records. Four trials evaluated secondary prevention populations, three trials evaluated primary prevention populations, and one trial evaluated a mixed population of individuals with and without established CHD. Many of the trials had design limitations, such as single-blinding, inclusion of electrocardiographically defined clinical endpoints, or open enrollment. All trials utilized blinded endpoint assessment. Quality scores were in the modest range and relatively homogeneous: all trials had quality scores of either 2 or 3. Combining all trials, the pooled risk reduction for CHD events was 19% (RR = 0.81, 95% CI 0.70–0.95, *p* = 0.008) (Figure 2). Statistical evidence for substantial between-study heterogeneity was not present (Q-statistic *p* = 0.13; I^2^ = 37%). In evaluating potential for publication bias, the trial by Watts et al. [8] was clearly a potential outlier both in terms of sample size and risk reduction. Excluding this trial, there was little change in the overall pooled result: RR = 0.82, 95% CI 0.70–0.95; *p* heterogeneity = 0.11, I^2^ = 42%. Visual inspection of the resulting funnel plot indicated some potential for publication bias (Figure S1), with a borderline Begg's test (continuity corrected *p* = 0.07), although such determinations are limited when the number of studies is relatively small.

**Table 1:** RCTs testing the effect on CHD events of increasing PUFA consumption in place of SFA.

Study	Population	PUFA Intake - Control (%E)	PUFA Intake – Intervention (%E)	Design	Intervention Strategy	Blinding	Dietary Assessment Method	Follow-up	No. of Events - Control	No. of Events - Intervention	CHD Outcome	Quality Score
Dayton 1968 – Los Angeles Veterans [1]	846 middle-aged and elderly semi-institutionalized men, with or without CHD	4.0^a^	14.9^a^	Parallel randomized	Partial feeding trial; ∼50% of meals eaten off-site	Double-blind	Direct analysis of provided foods	Up to 8 y	71	53	Total MI + SCD	3
Medical Research Council 1968 – Soy oil [2]	393 ambulatory men with recent MI	4.4^b^	20.4^b^	Parallel randomized	Dietary advice; emphasis on soybean oil	Blinded outcome assessment	Multiple serial weighed diet records	2–7 y	51	45	Total MI + SCD	2
Leren 1970 – Oslo Diet-Heart Study [3]	412 middle-aged ambulatory men with prior MI	5.2^c^	20.7	Parallel randomized	Dietary advice	Blinded outcome assessment	7 to 14 day weighed diet records in a subset	5 y^d^	81	61	Total MI + SCD	2
Turpeinen 1979 – Finnish Mental Hospital (men) [4]	∼461 middle-aged institutionalized men without CHD^e^	4.3	12.9	Cluster-randomized cross-over design, open enrollment^f^	Feeding trial; meals provided	Blinded outcome assessment	Direct analysis of provided foods	6 y in each arm	47	25	MI (assessed by major or intermediate ECG change) + CHD death	2
Miettinen 1983 – Finnish Mental Hospital (women) [5]	∼357 middle-aged institionalized women without CHD^e^	4.3	12.9	Cluster-randomized cross-over design, open enrollment^f^	Feeding trial; meals provided	Blinded outcome assessment	Direct analysis of provided foods	6 y in each arm	46	27	MI (assessed by major or intermediate ECG change) + CHD death	2
Frantz 1989 – Minnesota Coronary Survey [6]	9,057 institutionalized men and women without CHD	5.2	14.7	Parallel randomized, open enrollment	Feeding trial; meals provided	Double-blind	Direct analysis of provided foods	Average 1 y, max 4.5 y	121	131	Total MI + SCD	3
Burr 1989 – Diet and Reinfarction Trial [7]	2,033 ambulatory men with recent MI	6.4^b^	8.9^b^	Parallel randomized	Dietary advice	Blinded outcome assessment	Questionnaire validated against 7 day weighed diet records	2 y	144	132	MI + CHD death	2
Watts 1992 – St Thomas' Atherosclerosis Regression Study [8]	55 ambulatory men with established CHD	5.2^c^	8.0	Parallel randomized	Dietary advice; foods provided if requested	Blinded outcome assessment	Clinical interviews about dietary compliance	3.25 y	5	2	MI + death	2

a Linoleic acid consumption; total PUFA was not reported but would be very close.

b Calculated from published data in the trial on %E from total fat, the polyunsaturated∶saturated fat ratio, and type of intervention oil consumed, and plausible relative amounts of PUFA versus other fats based on the other trials.

c Imputed based upon the control diet in Frantz et al. (1989) that was also the median value among all control groups.

d Primary endpoint; post-hoc 11 year results not used.

e Results for incident CHD were reported among these participants without prevalent CHD. Results for total and cause-specific mortality were reported for all participants in a separate publication.

f The units of randomization were long-term-stay hospitals, and subjects joined the trial when they were hospitalized or exited when discharged.

ECG, electrocardiographic; MI, myocardial infarction; SCD, sudden cardiac death.

Weighted by the inverse-variance of each trial, the mean increase in PUFA consumption in the intervention group, compared to the control group, was 9.9%E, corresponding to a risk reduction for each 5%E greater PUFA consumption of 10% (RR = 0.90, 95% CI 0.83–0.97). Weighted by the inverse-variance of each trial, the mean decrease in blood total cholesterol (TC) levels in the intervention group, compared to the control group, was 0.76 mmol/l (29 mg/dl), corresponding to an observed risk reduction of 24% for each 1 mmol/l reduction in TC (RR = 0.76, 95% CI = 0.62–0.93).

The median duration of all trials was 4.25 years. Among the four trials with duration <4.25 years, the pooled RR was 0.91 (95% CI 0.76–1.10). Among the four trials with duration ≥4.25 years, the pooled RR was 0.73 (95% CI 0.61–0.87). In the four trials that evaluated exclusively or predominantly primary prevention populations, the pooled RR was 0.76 (95% CI 0.55–1.04). In the four trials that evaluated secondary prevention populations, the pooled RR was 0.84 (95% CI 0.72–0.98). For the six trials with a quality score of 2, the pooled RR was 0.78 (95% CI 0.66–0.91); for the two trials with a quality score of 3, the pooled RR was 0.91 (95% CI 0.63–1.31). Evaluating each of these potential sources of variation together in a metaregression model, study duration (*p* = 0.016), but not primary versus secondary prevention (*p* = 0.71) nor quality score (*p* = 0.78), was identified as a significant independent determinant of the extent of risk reduction. For each additional year of study duration, PUFA consumption lowered the relative risk of CHD events by an additional 9.2% in the intervention group (95% CI 1.7%–16.8%), compared with the control group. In secondary analyses restricted to CHD mortality alone (855 events, including 312 events from the full mortality report of one trial [34]), the pooled RR was 0.80 (95% CI 0.65–0.98). Evaluating total mortality due to all causes (2,472 events), the pooled RR was 0.98 (95% CI 0.89–1.08).

The overall pooled result for CHD events was not substantially altered in post-hoc secondary analyses based on specific study design characteristics. For example, excluding the three reports (two trials) with open enrollment, the overall pooled RR was 0.83 (95% CI 0.72–0.95, *p* = 0.006). Excluding the Finnish mental hospital trial (two reports) that used a cluster-randomization design, the overall pooled RR was 0.87 (95% CI 0.76–1.00, *p* = 0.05). Only two trials were double-blind; restricting to these two studies, the pooled RR was 0.91 (95% CI 0.63–1.31), with wide confidence intervals indicative of limited statistical power. Restricting to the four reports that provided meals (i.e., that were feeding trials), the pooled RR was 0.76 (95% CI 0.55–1.04, *p* = 0.08). Restricting to the four trials that provided mainly dietary advice, the pooled RR was 0.84 (95% CI 0.72–0.98, *p* = 0.03). None of these subgroup analyses were significantly different from the main pooled result, as demonstrated by the 95% CIs in each subgroup analysis including the value of the main pooled RR estimate of 0.81.

## Discussion

In this meta-analysis of RCTs, increasing PUFA consumption as a replacement for SFA reduced the occurrence of CHD events by 19%; each 5%E greater PUFA consumption reduced CHD risk by 10%. Whereas nearly all these trials were insufficiently powered to detect a significant effect individually, the pooled results demonstrate a significant benefit of replacing PUFA for SFA on clinical CHD events. Thus, this is only the second dietary intervention, together with consumption of long-chain omega-3 fatty acids (fish oil) [7],[35]–[37], that has now been clearly demonstrated to reduce cardiovascular events in RCTs.

**Figure 3:**
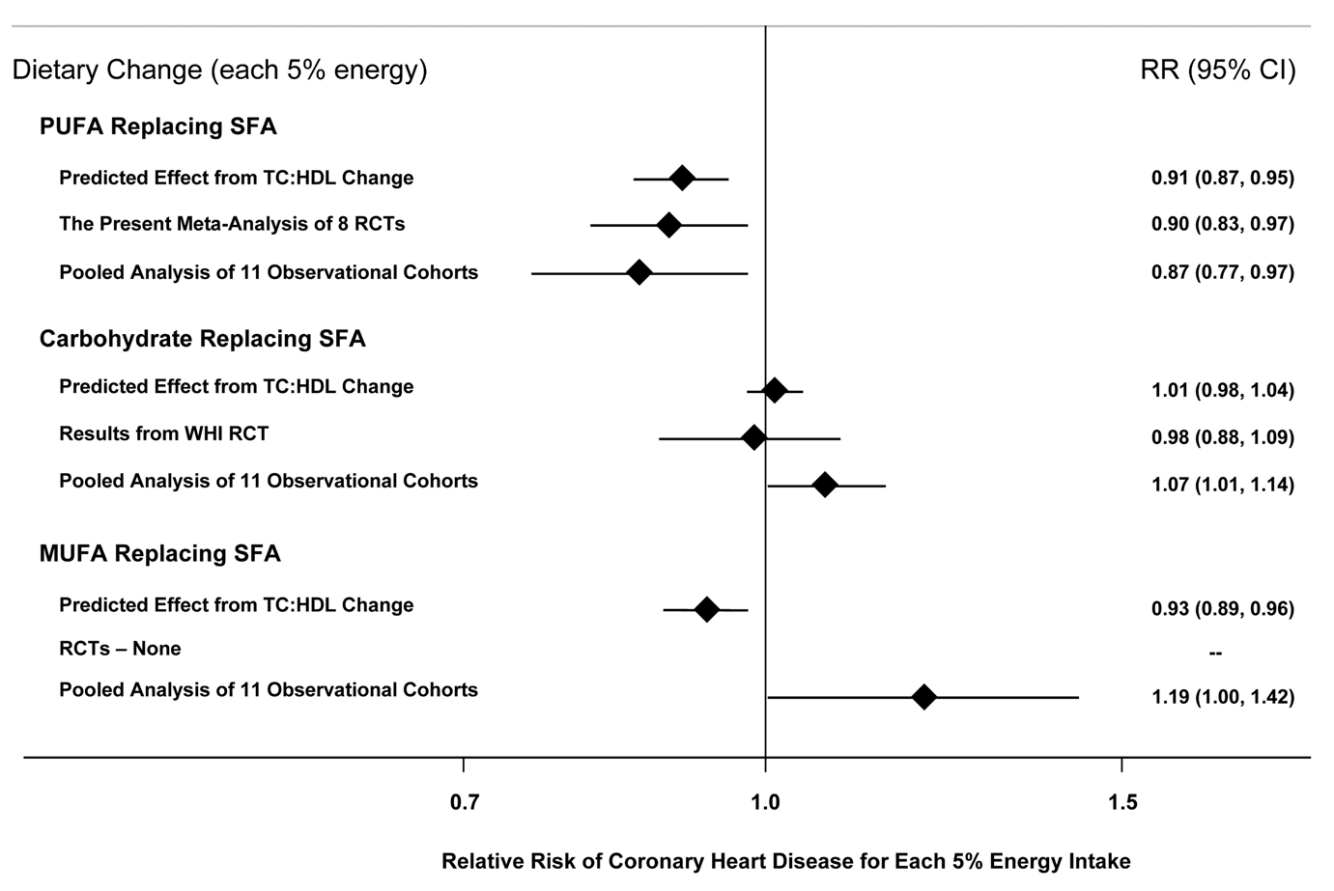
Effects on CHD risk of consuming PUFA, carbohydrate, or MUFA in place of SFA. Predicted effects are based on changes in the TC∶HDL-C ratio in short-term trials (e.g., each 5%E of PUFA replacing SFA lowers TC∶HDL-C ratio by 0.16) [11] coupled with observed associations between the TC∶HDL-C ratio and CHD outcomes in middle-aged adults (each 1 unit lower TC∶HDL-C is associated with 44% lower risk of CHD) [15]. Evidence for effects of dietary changes on actual CHD events comes from the present meta-analysis of eight RCTs for PUFA replacing SFA and from the Women's Health Initiative RCT for carbohydrate replacing SFA (*n* = 48,835, ∼3%E reduction in SFA over 8 years) [39]. Evidence for observed relationships of usual dietary habits with CHD events comes from a pooled analysis of 11 prospective cohort studies [38].

In short-term feeding trials, each 5%E of PUFA replacing SFA lowers low-density lipoprotein cholesterol (LDL-C) by 10 mg/dl, without an appreciable reduction in HDL-C, producing a lowering of the TC∶HDL-C ratio by 0.16; this can be compared to no significant change in the TC∶HDL-C ratio when SFA is replaced by carbohydrate [11]. In observational studies of adults aged 40–59 y, each 1 unit lower TC∶HDL-C is associated with 44% lower risk of CHD [15]. Based on these two sets of data, a 5%E increase in PUFA replacing SFA would be predicted, based on TC∶HDL-C effects alone, to reduce occurrence of CHD by 9% (Figure 3). Thus, the 10% risk reduction for a 5%E increase in PUFA replacing SFA demonstrated in the present meta-analysis of RCTs of clinical CHD outcomes is remarkably consistent with effects that would be predicted based on extension of the demonstrated lipid changes in short-term intervention trials to epidemiologic associations between TC∶HDL-C and CHD risk. A slightly greater risk reduction in studies of CHD events, compared with predicted effects based on lipid changes alone (Figure 3), is consistent with potential additional benefits of PUFA on other nonlipid pathways of risk such as insulin resistance [16],[17] and systemic inflammation [18]–[20]. Indeed, the impact of these additional benefits may be underestimated—the inevitable noncompliance in long-term dietary trials would attenuate true benefits, suggesting that the 10% risk reduction for a 5%E increase in PUFA in the present analysis may underestimate the full effects. Additionally, our analysis of heterogeneity indicates that longer-term trials showed greater benefits, suggesting that benefits of increasing PUFA consumption accrue over time.

When all trials were pooled, CHD risk was reduced by 24% for each 1 mmol/l reduction in TC (95% CI 7%–38%). This finding is consistent with results of observational studies of usual TC levels and CHD risk. In a pooled analysis from 61 prospective cohort studies including nearly 900,000 adults, each 1 mmol/l lower TC was associated with 28% lower risk of CHD death in adults aged 60–69 (RR = 0.72, 95% CI 0.69–0.74) and 42% lower risk of CHD death in adults aged 50–59 (RR = 0.58, 95% CI 0.56–0.61) [15], the ranges of ages included in the present trials. A comparison of our findings to those of long-term prospective observational studies of PUFA consumption is also informative. The most robust evidence to date comes from a recent report of pooled individual-level data from 11 cohort studies in America, Europe, and Israel, including 344,696 adults and 5,249 CHD events [38]. Each 5%E of greater PUFA consumption, as a replacement for SFA, was associated with 13% lower risk of CHD (RR = 0.87, 95% CI 0.77–0.97) (Figure 3). Our finding in RCTs of 10% lower risk of CHD for each 5%E of greater PUFA consumption, as a replacement for SFA, strongly supports both the causality and magnitude of these observational findings.

Because each of the RCTs in this meta-analysis tested the effects of consuming PUFA in place of SFA, the present findings cannot distinguish between potentially distinct benefits of increasing PUFA versus decreasing SFA. Thus, the present evidence alone is insufficient to conclude that increasing PUFA in place of any other nutrient will reduce CHD events. Notably, this evidence is similarly insufficient to conclude that decreasing SFA in place of any other nutrient will reduce CHD events. However, our findings indicate that a strategy of replacing SFA with PUFA is likely to reduce the occurrence of CHD.

Other lines of evidence—in particular, findings from RCTs of lipid risk factors and prospective cohort studies of CHD events—can provide insights into whether benefits may be more strongly related to reduced SFA, increased PUFA, or both. Based on either the predicted effects on TC∶HDL-C, the results of a large RCT [39], or a pooled analysis of 11 prospective cohort studies [38], replacement of SFA with carbohydrate does not lower CHD risk (Figure 3). Evidence for CHD effects of replacing SFA with monounsaturated fatty acids (MUFA) is mixed (Figure 3); randomized trials have not tested the effects of replacing SFA with MUFA. Thus, the evidence is most consistent and robust for CHD benefits when SFA is replaced with PUFA, rather than with MUFA or carbohydrate, suggesting that lower risk may be more strongly related to increased PUFA rather than decreased SFA consumption. Recent ecological studies across nations over time also support this contention, with changes in population CHD mortality being most strongly related to increased consumption of vegetable oils that contained PUFA, particularly the n-3 PUFA alpha-linolenic acid, rather than decreases in animal fats or increases in overall vegetable consumption [40]. Further studies are needed to evaluate the role of MUFA or protein as a replacement for other macronutrients on risk of CHD.

The eight trials in this meta-analysis were performed and reported with a relatively regular distribution over nearly three decades between 1968 and 1992. This broad time span could increase generalizability, and there is likely little reason to believe that the biologic effects of PUFA have changed in recent years. The use of random-effects meta-analysis allowed the pooling and estimation of overall variance of different trials that may also each be estimating a different “true” effect. All of these RCTs had blinded endpoint ascertainment that would limit the magnitude of potential differential (biased) assignment of types of events or causes of death.

Many of the identified randomized trials in our meta-analysis had important design limitations (Table 1). For example, some trials provided all or most meals, increasing compliance but perhaps limiting generalizability to effects of dietary recommendations alone; whereas other trials relied only on dietary advice, increasing generalizability to dietary recommendations but likely underestimating efficacy due to noncompliance. Several of these trials were not double-blind, raising the possibility of differential classification of endpoints by the investigators that could overestimate benefits of the intervention. One trial used a cluster-randomization cross-over design that intervened on sites rather than individuals; and two trials used open enrollment that allowed participants to both drop-in and drop-out during the trial. The methods for estimating and reporting PUFA and SFA consumption in each trial varied, which could cause errors in our estimation of the quantitative benefit per %E replacement. One of the trials also provided, in addition to the main advice to consume soybean oil, sardines to the intervention group [3], so that observed benefits may be at least partly related to marine omega-3 PUFA rather than total PUFA consumption. Several of the trials specified use of vegetable oils containing, in addition to omega-6 PUFA, small amounts of the omega-3 PUFA alpha-linolenic acid [2]–[5],[8], although additional benefits of this plant-derived omega-3, compared with seafood-derived omega-3, are not yet clearly established [41].

Given these limitations of each individual trial, the quantitative pooled risk estimate should be interpreted with some caution. Nevertheless, this is the best current worldwide evidence from RCTs for effects on CHD events of replacing SFA with PUFA, and, as discussed above, the pooled risk estimate from this meta-analysis (10% lower risk per 5%E greater PUFA) is well within the range of estimated benefits from randomized controlled feeding trials of changes in lipid levels (9% lower risk per 5%E greater PUFA) and prospective observational studies of clinical CHD events (13% lower risk per 5%E greater PUFA). The consistency of the findings across these different lines of evidence provides substantial confidence in both the qualitative benefits and also a fairly narrow range of quantitative uncertainty.

As in any meta-analysis, publication bias is a potential limitation. It seems unlikely that large dietary clinical trials would have been performed and not reported without any knowledge of the community of experts, and if smaller trials were performed and unpublished, their addition would be unlikely to substantially alter the pooled risk estimate given the numbers of subjects and events currently included. Additionally, our direct contact with experts minimized the possibility of missing unpublished studies. The findings of this meta-analysis cannot be extrapolated to effects of replacing SFA with carbohydrate or MUFA (Figure 3), which were not evaluated in the present trials. Results should also not be extrapolated to effects of increasing PUFA as replacement for carbohydrate, although based on changes in TC∶HDL-C in feeding studies [11], and observed relationships with clinical events in cohort studies [38],[42], one would predict CHD benefit from such replacement. Future trials should investigate these other dietary interventions, in particular increasing PUFA consumption as a replacement for carbohydrate and also MUFA.

This current meta-analysis of RCTs of clinical CHD events, together with consistent findings from both prospective cohort studies of clinical CHD events and RCTs of intermediate risk factors, provides strong concordant evidence that consumption of PUFA, in place of SFA, lowers CHD risk. Our findings have several immediate implications. First, our results, together with data from other research paradigms discussed above, indicate that evidence-based population- and individual-level recommendations to reduce SFA consumption should specify the importance of replacement with PUFA. Second, because many of these trials used vegetable oils containing small amounts of plant-derived n-3 PUFA in addition to omega-6 PUFA, our findings as well as those of ecologic studies [40] would support focus on n-3 PUFA-containing vegetable oils, such as soybean or canola, to increase population PUFA intake. For example, daily consumption of 20 g soybean oil or 30 g canola oil, as an isocaloric replacement for other macronutrients, would increase PUFA consumption by ∼5%E on a 2000 kcal/d diet [43]. Third, our findings demonstrate reductions in CHD events, and no evidence for increased risk, in long-term trials utilizing PUFA consumption at very high levels (mean = 14.9%E, range 8.0%E –20.7%E). This suggests that current recommendations for an upper limit of PUFA consumption at 10%E [12]–[14] need to be revisited, particularly as PUFA appears to be the primary evidence-based replacement for SFA. Finally, whereas on a population-level even a small shift from SFA to PUFA consumption would produce meaningful reductions in CHD risk, the relatively modest magnitude of plausible benefit (∼10% lower risk for 5%E replacement) indicates a need for substantial policy focus on other dietary risk factors for CHD [44], in particular high consumption of salt and low consumption of seafood, whole grains, fruits, and vegetables.

## Supporting Information

Figure S1 Funnel plot of the log-relative risks (beta) versus their standard error (s.e. of beta).
(0.08 MB TIF)

Text S1 PRISMA statement.
(0.08 MB DOC)

Text S2 Protocol.
(0.09 MB DOC)

Table S1 List of excluded studies.
(0.14 MB DOC)

## Abbreviations

%E: percent energy
CHD: coronary heart disease
CI: confidence interval
HDL-C: high-density lipoprotein cholesterol
LDL-C: low-density lipoprotein-cholesterol
MUFA: monounsaturated fat
PUFA: polyunsaturated fat
RCT: randomized controlled trial
RR: risk ratio
SE: standard error
SFA: saturated fat
TC: total cholesterol
