# Supplementary figures and images for: Effects on Coronary Heart Disease of Increasing Polyunsaturated Fat in Place of Saturated Fat: A Systematic Review and Meta-Analysis of Randomized Controlled Trials
Source: PLoS Med. 2010 Mar 23;7(3):e1000252. doi: 10.1371/journal.pmed.1000252 (PMC2843598; doi:10.1371/journal.pmed.1000252)

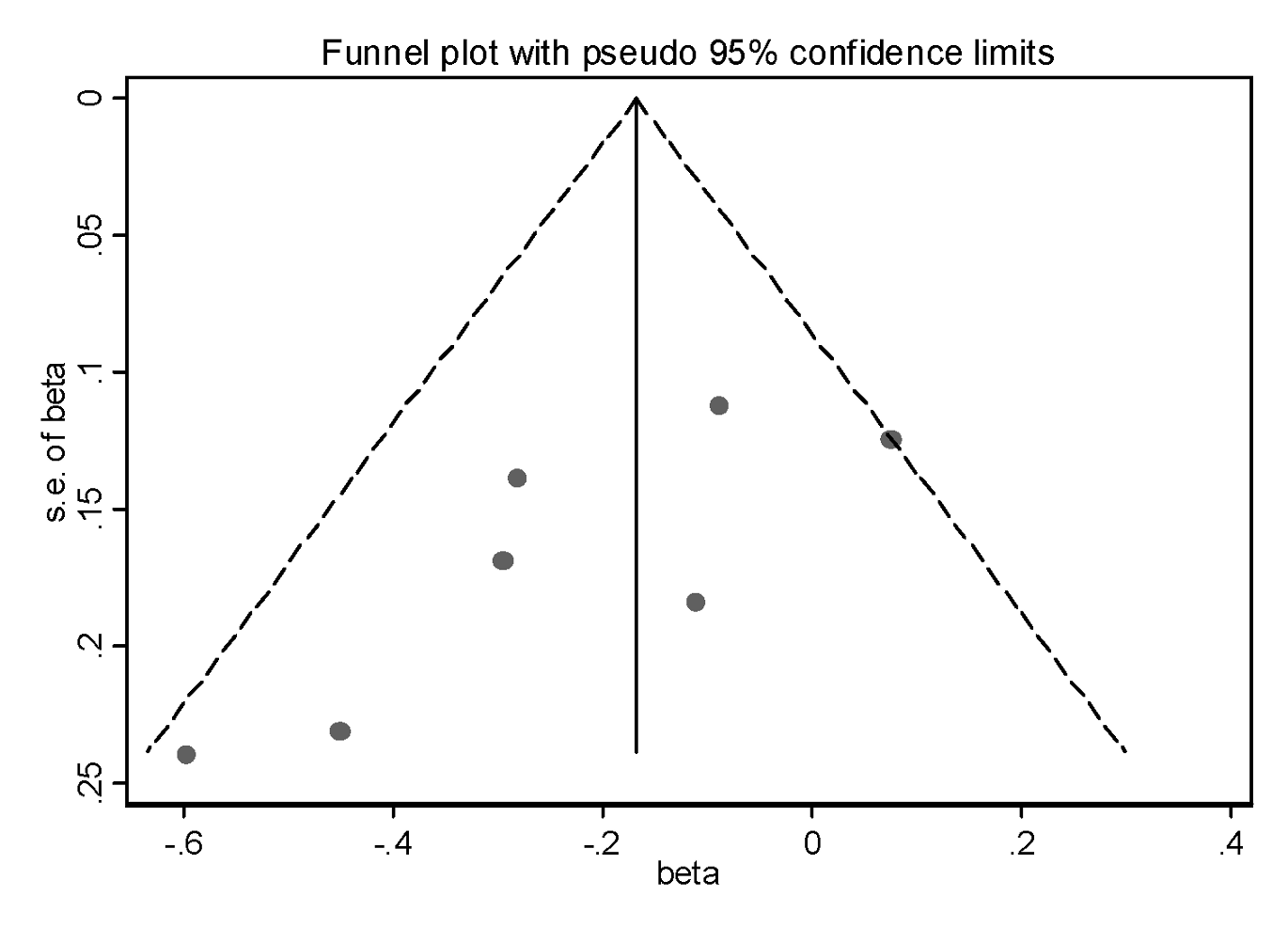

Supplement: Figure S1 — Funnel plot of the log-relative risks (beta) versus their standard error (s.e. of beta). (0.08 MB TIF) [file pmed.1000252.s001.tif]
