# Supplementary material for: Effects on Coronary Heart Disease of Increasing Polyunsaturated Fat in Place of Saturated Fat: A Systematic Review and Meta-Analysis of Randomized Controlled Trials
Source: PLoS Med. 2010 Mar 23;7(3):e1000252. doi: 10.1371/journal.pmed.1000252 (PMC2843598; doi:10.1371/journal.pmed.1000252)
Supplement: Text S2 — Protocol. (0.09 MB DOC) [file pmed.1000252.s003.doc]

**METHODS**

The recommendations improving the quality of reports of Meta-analysis of randomized controlled trials (QUOROM)[1]guidelines were followed during all stages of the design, implementation, and reporting of this meta-analysis.

**Search strategy:**

- **Inclusion criteria**
  1. All randomized controlled trials that randomized adults to increased total or n-6 PUFA consumption for at least 1 year without other major concomitant interventions (e.g., blood pressure control, smoking control, other multiple dietary interventions, etc.), had an appropriate control group without this dietary intervention, and reported effects on occurrence of “hard” CHD events (myocardial infarction, CHD death, and/or sudden death).
  2. The studies report (or can be obtained from authors) risk estimates with standard errors (or information to compute these) for CHD events.
- **Exclusion criteria**

1. Observational or other non-randomized studies.
2. Studies in which the intervention was mainly n-3 PUFA.
3. Studies only having information on secondary endpoints (e.g., lipid levels) or “soft” CHD outcomes (e.g., angina).
4. Commentaries or reviews
5. Duplicate publications from the same study.

- **Databases searched**

1. PUBMED
2. EMBASE
3. The Cochrane library
4. Agris, Amed, HMIC, PsycINFO
5. WEB OF KNOWLEDGE
   - - BIOSIS
     - WEB OF SCIENCE
     - ISI proceedings
6. CINAHL
7. CAB abstracts (http://www.cabi.org/)
8. Conference abstracts (ZETOC)
9. Faculty of 1000
10. Grey literature sources (SIGLE; system for information on grey literature in Europe, British library inside database, and dissertation abstracts online)
11. Related articles
12. Hand searching reference lists of identified studies

**PUBMED SEARCH**

**Limits:** Years to be searched included the earliest available online year of indexing up to now without language restrictions. Limit to humans, adults only, and randomized controlled trials.

**SEARCH CARRIED OUT ON 22/JUNE/2009**

- **Search query for Pubmed**

**PUFA query**

("Fatty Acids, Omega-6"[Mesh] OR “unsaturated fatty acid”[tiab] OR “unsaturated fatty acids”[tiab] OR “unsaturated fat”[tiab] OR “unsaturated fats”[tiab] OR “polyunsaturated fatty acid”[tiab] OR “polyunsaturated fatty acids”[tiab] OR “polyunsaturated fat”[tiab] OR “polyunsaturated fats”[tiab] OR “omega-6”[tiab] OR “linoleic”[tiab] OR “octadecadienoic acid”[tiab] OR “safflower oil”[tiab] OR “sesame oil”[tiab] OR “soybean oil”[tiab] OR “soyabean oil”[tiab] OR “corn oil”[tiab])

**Diseases query**

**AND** ("cardiovascular diseases"[Mesh] OR "cardiovascular disease"[tiab] OR "cardiovascular diseases"[tiab] OR "heart disease"[tiab] OR "heart diseases"[tiab] OR "myocardial infarction"[tiab] OR "myocardial infarctions"[tiab] OR "heart attack"[tiab] OR "heart attacks"[tiab] OR "sudden death"[tiab] OR "sudden deaths"[tiab] OR "coronary syndrome"[tiab])

**Publication type or STROKE OR n-3**

**NOT** ("Case Reports"[Publication Type] OR "stroke"[tiab] OR "strokes"[tiab] OR "cerebrovascular accident"[tiab] OR "cerebrovascular accidents"[tiab] OR “Fatty Acids, Omega-3”[Mesh] OR “omega-3”[tw] OR “n-3”[tw])

**All together**

("Fatty Acids, Omega-6"[Mesh] OR “unsaturated fatty acid”[tiab] OR “unsaturated fatty acids”[tiab] OR “unsaturated fat”[tiab] OR “unsaturated fats”[tiab] OR “polyunsaturated fatty acid”[tiab] OR “polyunsaturated fatty acids”[tiab] OR “polyunsaturated fat”[tiab] OR “polyunsaturated fats”[tiab] OR “omega-6”[tiab] OR “linoleic”[tiab] OR “octadecadienoic acid”[tiab] OR “safflower oil”[tiab] OR “sesame oil”[tiab] OR “soybean oil”[tiab] OR “soyabean oil”[tiab] OR “corn oil”[tiab]) **AND** ("cardiovascular diseases"[Mesh] OR "cardiovascular disease"[tiab] OR "cardiovascular diseases"[tiab] OR "heart disease"[tiab] OR "heart diseases"[tiab] OR "myocardial infarction"[tiab] OR "myocardial infarctions"[tiab] OR "heart attack"[tiab] OR "heart attacks"[tiab] OR "sudden death"[tiab] OR "sudden deaths"[tiab] OR "coronary syndrome"[tiab]) **NOT** ("Case Reports"[Publication Type] OR "stroke"[tiab] OR "strokes"[tiab] OR "cerebrovascular accident"[tiab] OR "cerebrovascular accidents"[tiab] OR “Fatty Acids, Omega-3”[Mesh] OR “omega-3”[tw] OR “n-3”[tw])

**HITS (limited to humans, all adult, and clinical trials, randomized controlled trials, and meta-analyses): 181**

- **Search query for OVID (free text): Embase, Agris, Amed: http://gateway.ovid.com/autologin.cgi**

(("polyunsaturated fatty acid" OR "polyunsaturated fatty acids" OR "polyunsaturated fat" OR "polyunsaturated fats" OR "unsaturated fatty acid" OR "unsaturated fatty acids" OR "unsaturated fat" OR "unsaturated fats" OR "omega-6" OR "linoleic" OR "octadecadienoic acid") **AND** ("cardiovascular disease" OR "cardiovascular diseases" OR "heart disease" OR "heart diseases" OR "myocardial infarction" OR "myocardial infarctions" OR "heart attack" OR "heart attacks" OR "sudden death" OR "sudden deaths" OR "coronary syndrome") **AND** ("trial" OR "trials"))

**Hits: 185 limits: humans, all adult, remove duplicates** (import option for ovid: AARP Ageline (OVID)**)**

- **Search query for Web of knowledge (3 databases):**

**http://apps.isiknowledge.com/**

("polyunsaturated fatty acid" OR "polyunsaturated fatty acids" OR "polyunsaturated fat" OR "polyunsaturated fats" OR "unsaturated fatty acid" OR "unsaturated fatty acids" OR "unsaturated fat" OR "unsaturated fats" OR "omega-6" OR "n-6" OR "linoleic" OR "octadecadienoic acid") AND Topic=("cardiovascular disease" OR "cardiovascular diseases" OR "heart disease" OR "heart diseases" OR "myocardial infarction" OR "myocardial infarctions" OR "heart attack" OR "heart attacks" OR "sudden death" OR "sudden deaths" OR "coronary syndrome") AND Topic=("trial" OR "trials") NOT Topic=("case report" OR "case control" OR "cohort" OR "prospective" OR "longitudinal")

**Hits: 9 (limits: search in document types: clinical trial)**

- **Search query for Other databases (simpler search strategy)**

("polyunsaturated fatty acid" OR "polyunsaturated fatty acids" OR "polyunsaturated fat" OR "polyunsaturated fats" OR "unsaturated fatty acid" OR "unsaturated fatty acids" OR "unsaturated fat" OR "unsaturated fats" OR "omega-6" OR "n-6" OR "linoleic") AND (Cardiovascular disease OR cardiovascular diseases OR coronary heart disease OR myocardial infarction OR heart attack)

**Hits: 0 (Cochrane library) (limits: not possible to apply limits)**

**Faculty of 1,000: 6 (limits: not possible to apply limits)**

**After combining searches and removing duplicates: total N1: 346**

- **Study selection process**
  - 1 investigator reviewed titles/ abstracts.
  - 2 investigators separately reviewed full-text for inclusion/ exclusions and performed data abstraction in duplicate.
  - Inter-observer agreement between the two reviewers on study inclusion was 96% (for 2/54 screened full-text, there was initial discordance) .
  - Disagreement was resolved by mutual discussion and if, required, by consultation with a third investigator.

**Data extraction**

- Quality assessment based on a quality score (Jadad quality score[2], based on 3 factors)
- Using a standardized data extraction sheet information was extracted on the study and in duplicate, including:
  - Author
  - Study name
  - Year of publication
  - Years (dates) study was performed
  - Duration of follow-up
  - Study design
  - Study location
  - Exposure definition (e.g., total PUFA, LA, n-6)
  - Population
  - Age of participants (mean, SD, range)
  - Control diet
  - Intervention diet
  - Intake of PUFA in the control group (as % kcal)
  - Intake of PUFA in the intervention group (as % kcal)
  - Absolute change in total cholesterol (in mg/dl)
  - Percent change in total cholesterol
  - No of subjects in the control group
  - No of subjects in the intervention group
  - No of events in the control group
  - No of events in the intervention group
  - Outcome CHD (which one)
  - Relative ratio, betas, SE, and corresponding confidence interval (either reported or directly calculated)
- Authors of published studies were contacted to request missing data

References

1. Moher, D., et al., *Improving the quality of reports of meta-analyses of randomised controlled trials: the QUOROM statement. QUOROM Group.* Br J Surg, 2000. **87**(11): p. 1448-54.

2. Jadad, A.R., et al., *Assessing the quality of reports of randomized clinical trials: is blinding necessary?* Control Clin Trials, 1996. **17**(1): p. 1-12.
