# Supplementary material for: Effects on Coronary Heart Disease of Increasing Polyunsaturated Fat in Place of Saturated Fat: A Systematic Review and Meta-Analysis of Randomized Controlled Trials
Source: PLoS Med. 2010 Mar 23;7(3):e1000252. doi: 10.1371/journal.pmed.1000252 (PMC2843598; doi:10.1371/journal.pmed.1000252)
Supplement: Table S1 — List of excluded studies. (0.14 MB DOC) [file pmed.1000252.s004.doc]

**Supporting Information Table. List of the 46 excluded studies and reasons for exclusion.**

| **Study** | **Reason for Exclusion** |
| --- | --- |
| 1. (Hu and Willett 2002) | Review |
| 1. (Ravnskov 1998) | Review |
| 1. (Hebert, Gaziano et al. 1995) | Review |
| 1. (Dolecek and Granditis 1991) | Observational study |
| 1. (Dolecek 1992) | Observational study |
| 1. (Kardinaal, Aro et al. 1995) | Observational study |
| 1. (Bemelmans, Muskiet et al. 2000) | Observational study |
| 1. (Blankenhorn, Johnson et al. 1990) | Observational study |
| 1. (Pearce and Dayton 1971) | Duplicate publication |
| 1. (Watts, Lewis et al. 1995) | Duplicate publication |
| 1. (Miettinen, Turpeinen et al. 1972) | Duplicate publication |
| 1. (Blann, Jackson et al. 1995) | Duplicate publication |
| 1. (Hartman 1995) | Duplicate publication |
| 1. (Gorder, Dolecek et al. 1986) | Duplicate publication |
| 1. (Dayton and Pearce 1969) | Duplicate publication |
| 1. (Dolecek, Bradham et al. 1996) | Duration <1 year |
| 1. (Ferrara, Raimondi et al. 2000) | Duration <1 year |
| 1. (Higdon, Du et al. 2001) | Duration <1 year |
| 1. (Moore, Bryant et al. 2006) | Duration <1 year |
| 1. (Zhao, Etherton et al. 2004) | Duration <1 year |
| 1. (Zock, Mensink et al. 1997) | Duration <1 year |
| 1. (Bemelmans, Broer et al. 2002) | Not a trial of increased PUFA vs. control |
| 1. (Borchgrevink, Skaga et al. 1966) | Not a trial of increased PUFA vs. control |
| 1. (Leng, Lee et al. 1998) | Not a trial of increased PUFA vs. control |
| 1. (Ghafoorunissa, Vani et al. 2002) | Not a trial of increased PUFA vs. control |
| 1. (Grundt, Nilsen et al. 2003) | Not a trial of increased PUFA vs. control |
| 1. (Natvig 1967) | Not a trial of increased PUFA vs. control |
| 1. (Dodin, Cunnane et al. 2008) | Not a trial of increased PUFA vs. control |
| 1. (De Lorgeril, Renaud et al. 1994) | Multiple interventions |
| 1. (De Lorgeril, Salen et al. 1998) | Multiple interventions |
| 1. (Esposito, Marfella et al. 2004) | Multiple interventions |
| 1. (Howard, Van Horn et al. 2006) | Multiple interventions |
| 1. (Zazpe, Sanchez-Tainta et al. 2008) | Multiple interventions |
| 1. (Renaud, De Lorgeril et al. 1995) | Multiple interventions |
| 1. (Singh, Rastogi et al. 1991) | Multiple interventions |
| 1. (Committee 1965) | Multiple interventions |
| 1. (Strandberg, Salomaa et al. 1991) | Multiple interventions |
| 1. (Organisation 1986) | Multiple interventions |
| 1. (Investigators 1990) | Multiple interventions |
| 1. (Hjermann, Velve Byre et al. 1981) | Multiple interventions |
| 1. (Singh, Rastogi et al. 1992) | Multiple interventions |
| 1. (Rose, Thomson et al. 1965) | Multiple interventions |
| 1. (Probstfield and Rifkind 1991) | Multiple interventions |
| 1. (Howard-Williams, Patel et al. 1985) | Non-CHD endpoint |
| 1. (Woodhill, Palmer et al. 1978) | Non-CHD endpoint |
| 1. (Bierenbaum, Fleischman et al. 1970) | Non-randomized |

**References**

Bemelmans, W. J. E., J. Broer, et al. (2002). Effect of an increased intake of alpha-linolenic acid and group nutritional education on cardiovascular risk factors: The Mediterranean Alpha-linolenic Enriched Groningen Dietary Intervention (MARGARIN) study, American Journal of Clinical Nutrition. 75(2)(pp 221-227), 2002. Date of Publication: 2002.

Bemelmans, W. J. E., F. A. J. Muskiet, et al. (2000). Associations of alpha-linolenic acid and linoleic acid with risk factors for coronary heart disease, European Journal of Clinical Nutrition. 54(12)(pp 865-871), 2000. Date of Publication: 2000.

Bierenbaum, M. L., A. I. Fleischman, et al. (1970). "The 5-year experience of modified fat diets on younger men with coronary heart disease." Circulation **42**(5): 943-52.

Blankenhorn, D. H., R. L. Johnson, et al. (1990). "The influence of diet on the appearance of new lesions in human coronary arteries." Jama **263**(12): 1646-52.

Blann, A. D., P. Jackson, et al. (1995). "von Willebrand factor, a possible indicator of endothelial cell damage, decreases during long-term compliance with a lipid-lowering diet." J Intern Med **237**(6): 557-61.

Borchgrevink, C. F., E. Skaga, et al. (1966). "Absence of prophylactic effect of linolenic acid in patients with coronary heart-disease." Lancet **2**(7456): 187-9.

Committee, R. (1965). "Low-fat diet in myocardial infarction: A controlled trial." Lancet **2**(7411): 501-4.

Dayton, S. and M. L. Pearce (1969). "Diet high in unsaturated fat. A controlled clinical trial." Minn Med **52**(8): 1237-42.

De Lorgeril, M., S. Renaud, et al. (1994). Mediterranean alpha-linolenic acid-rich diet in secondary prevention of coronary heart disease, Lancet. 343(8911)(pp 1454-1459), 1994. Date of Publication: 1994.

De Lorgeril, M., P. Salen, et al. (1998). Mediterranean dietary pattern in a randomized trial: Prolonged survival and possible reduced cancer rate, Archives of Internal Medicine. 158(11)(pp 1181-1187), 1998. Date of Publication: 08 Jun 1998.

Dodin, S., S. C. Cunnane, et al. (2008). Flaxseed on cardiovascular disease markers in healthy menopausal women: a randomized, double-blind, placebo-controlled trial, Nutrition. 24(1)(pp 23-30), 2008. Date of Publication: Jan 2008.

Dolecek, T. A. (1992). Epidemiological evidence of relationships between dietary polyunsaturated fatty acids and mortality in the Multiple Risk Factor Intervention Trial, Proceedings of the Society for Experimental Biology and Medicine. 200(2)(pp 177-182), 1992. Date of Publication: 1992.

Dolecek, T. A., K. H. Bradham, et al. (1996). "Maximizing recruitment efforts in a drug lipid-lowering trial with dietary intervention to lower LDL cholesterol." Control Clin Trials **17**(1): 33-45.

Dolecek, T. A. and G. Granditis (1991). "Dietary polyunsaturated fatty acids and mortality in the Multiple Risk Factor Intervention Trial (MRFIT)." World Rev Nutr Diet **66**: 205-16.

Esposito, K., R. Marfella, et al. (2004). Effect of a Mediterranean-style diet on endothelial dysfunction and markers of vascular inflammation in the metabolic syndrome: A randomized trial, Journal of the American Medical Association. 292(12)(pp 1440-1446), 2004. Date of Publication: 22 Sep 2004.

Ferrara, L. A., A. S. Raimondi, et al. (2000). "Olive oil and reduced need for antihypertensive medications." Arch Intern Med **160**(6): 837-42.

Ghafoorunissa, A. Vani, et al. (2002). Effects of dietary alpha-linolenic acid from blended oils on biochemical indices of coronary heart disease in Indians, Lipids. 37(11)(pp 1077-1086), 2002. Date of Publication: 01 Nov 2002.

Gorder, D. D., T. A. Dolecek, et al. (1986). "Dietary intake in the Multiple Risk Factor Intervention Trial (MRFIT): nutrient and food group changes over 6 years." J Am Diet Assoc **86**(6): 744-51.

Grundt, H., D. W. T. Nilsen, et al. (2003). Reduction in homocysteine by n-3 polyunsaturated fatty acids after 1 year in a randomised double-blind study following an acute myocardial infarction: No effect on endothelial adhesion properties, Pathophysiology of Haemostasis and Thrombosis. 33(2)(pp 88-95), 2003. Date of Publication: 2003.

Hartman, I. S. (1995). Alpha-linolenic acid: A preventive in secondary coronary events?, Nutrition Reviews. 53(7)(pp 194-197), 1995. Date of Publication: 1995.

Hebert, P. R., J. M. Gaziano, et al. (1995). An overview of trials of cholesterol lowering and risk of stroke, Archives of Internal Medicine. 155(1)(pp 50-55), 1995. Date of Publication: 09 Jan 1995.

Higdon, J. V., S. H. Du, et al. (2001). Supplementation of postmenopausal women with fish oil does not increase overall oxidation of LDL ex vivo compared to dietary oils rich in oleate and linoleate, Journal of Lipid Research. 42(3)(pp 407-418), 2001. Date of Publication: 2001.

Hjermann, I., K. Velve Byre, et al. (1981). "Effect of diet and smoking intervention on the incidence of coronary heart disease. Report from the Oslo Study Group of a randomised trial in healthy men." Lancet **2**(8259): 1303-10.

Howard-Williams, J., P. Patel, et al. (1985). "Polyunsaturated fatty acids and diabetic retinopathy." Br J Ophthalmol **69**(1): 15-8.

Howard, B. V., L. Van Horn, et al. (2006). Low-fat dietary pattern and risk of cardiovascular disease: The Women's Health Initiative randomized controlled dietary modification trial, Journal of the American Medical Association. 295(6)(pp 655-666), 2006. Date of Publication: 08 Feb 2006.

Hu, F. B. and W. C. Willett (2002). Optimal diets for prevention of coronary heart disease, Journal of the American Medical Association. 288(20)(pp 2569-2578), 2002. Date of Publication: 27 Nov 2002.

Investigators, M. t. (1990). "Mortality rates after 10.5 years for participants in the Multiple Risk Factor Intervention Trial. Findings related to a priori hypotheses of the trial. The Multiple Risk Factor Intervention Trial Research Group." Jama **263**(13): 1795-801.

Kardinaal, A. F., A. Aro, et al. (1995). "Association between beta-carotene and acute myocardial infarction depends on polyunsaturated fatty acid status. The EURAMIC Study. European Study on Antioxidants, Myocardial Infarction, and Cancer of the Breast." Arterioscler Thromb Vasc Biol **15**(6): 726-32.

Leng, G. C., A. J. Lee, et al. (1998). Randomized controlled trial of gamma-linolenic acid and eicosapentaenoic acid in peripheral arterial disease, Clinical Nutrition. 17(6)(pp 265-271), 1998. Date of Publication: 1998.

Miettinen, M., O. Turpeinen, et al. (1972). "Effect of cholesterol-lowering diet on mortality from coronary heart-disease and other causes. A twelve-year clinical trial in men and women." Lancet **2**(7782): 835-8.

Moore, C. S., S. P. Bryant, et al. (2006). Oily fish reduces plasma triacylglycerols: a primary prevention study in overweight men and women, Nutrition. 22(10)(pp 1012-1024), 2006. Date of Publication: Oct 2006.

Natvig, H. (1967). "[The effect of unsaturated fatty acids on the incidence of coronary infarction, etc.]." Tidsskr Nor Laegeforen **87**(11): 1033-41.

Organisation, W. H. (1986). "European collaborative trial of multifactorial prevention of coronary heart disease: final report on the 6-year results. World Health Organisation European Collaborative Group." Lancet **1**(8486): 869-72.

Pearce, M. L. and S. Dayton (1971). "Incidence of cancer in men on a diet high in polyunsaturated fat." Lancet **1**(7697): 464-7.

Probstfield, J. L. and B. M. Rifkind (1991). The Lipid Research Clinics Coronary Primary Prevention Trial: Design, results, and implications, European Journal of Clinical Pharmacology. 40(SUPPL. 1)(pp S69-S75), 1991. Date of Publication: 1991.

Ravnskov, U. (1998). The questionable role of saturated and polyunsaturated fatty acids in cardiovascular disease, Journal of Clinical Epidemiology. 51(6)(pp 443-460), 1998. Date of Publication: Jun 1998.

Renaud, S., M. De Lorgeril, et al. (1995). Cretan Mediterranean diet for prevention of coronary heart disease, American Journal of Clinical Nutrition. 61(6 SUPPL.)(pp 1360S-1367S), 1995. Date of Publication: Jun 1995.

Rose, G. A., W. B. Thomson, et al. (1965). "Corn Oil in Treatment of Ischaemic Heart Disease." Br Med J **1**(5449): 1531-3.

Singh, R. B., S. S. Rastogi, et al. (1991). "Dietary strategies for risk-factor modification to prevent cardiovascular diseases." Nutrition **7**(3): 210-4.

Singh, R. B., S. S. Rastogi, et al. (1992). "Randomised controlled trial of cardioprotective diet in patients with recent acute myocardial infarction: results of one year follow up." Bmj **304**(6833): 1015-9.

Strandberg, T. E., V. V. Salomaa, et al. (1991). "Long-term mortality after 5-year multifactorial primary prevention of cardiovascular diseases in middle-aged men." Jama **266**(9): 1225-9.

Watts, G. F., B. Lewis, et al. (1995). "Relationships between nutrient intake and progression/regression of coronary atherosclerosis as assessed by serial quantitative angiography." Can J Cardiol **11 Suppl G**: 110G-114G.

Woodhill, J. M., A. J. Palmer, et al. (1978). "Low fat, low cholesterol diet in secondary prevention of coronary heart disease." Adv Exp Med Biol **109**: 317-30.

Zazpe, I., A. Sanchez-Tainta, et al. (2008). "A large randomized individual and group intervention conducted by registered dietitians increased adherence to Mediterranean-type diets: the PREDIMED study." J Am Diet Assoc **108**(7): 1134-44; discussion 1145.

Zhao, G., T. D. Etherton, et al. (2004). Dietary alpha-linolenic acid reduces inflammatory and lipid cardiovascular risk factors in hypercholesterolemic men and women, Journal of Nutrition. 134(11)(pp 2991-2997), 2004. Date of Publication: Nov 2004.

Zock, P. L., R. P. Mensink, et al. (1997). Fatty acids in serum cholesteryl esters as quantitative biomarkers of dietary intake in humans, American Journal of Epidemiology. 145(12)(pp 1114-1122), 1997. Date of Publication: 15 Jun 1997.
